# New Arrangements in the Medical Department of the University of Buffalo

**Published:** 1859-03

**Authors:** 


*New Appointments in the Medical Department of the University of Buffalo.*—Dr. Sanford Eastman, who was appointed last year lecturer on Anatomy in the Buffalo Medical College, has been appointed professor of this branch.

The editor of this Journal has also been honored by the appointment to the professorship of Physiology and Microscopic Anatomy.

